# A double-blind placebo needle for acupuncture research

**DOI:** 10.1186/1472-6882-7-31

**Published:** 2007-10-10

**Authors:** Nobuari Takakura, Hiroyoshi Yajima

**Affiliations:** 1 Hanada College: Japan School of Acupuncture, Moxibustion and Physiotherapy 20-1 Sakuragaoka-machi, Shibuya-ku, Tokyo 150-0031, Japan; 2 Second Department of Physiology, Showa University School of Medicine, 1-5-8 Hatanodai, Shinagawa-ku, Tokyo 142-8555, Japan; 3 The Institute for Oriental Medicine Research Foundation, 28-9 Sakuragaoka-machi, Shibuya-ku, Tokyo 150-0031, Japan

## Abstract

**Background:**

Placebo needles that can mask acupuncture practitioners to the type of needle used have been considered almost impossible to develop until now.

**Methods:**

We designed a double-blind non-penetrating placebo needle, the needle tip of which simply presses against the skin, and a matched penetrating needle. The needles are encased inside an opaque guide tube and the appearance and feel of the pair are designed to be indistinguishable. To validate the masking effect for the practitioner, 10 acupuncturists each applied 23 non-penetrating needles and 17 penetrating needles to the Large Intestine-4 point. After removing each needle, they judged whether the needle was 'penetrating', 'non-penetrating' or 'unidentifiable'. For the validation of patient masking, an acupuncturist randomly applied a non-penetrating/penetrating needle pair to the bilateral Sanjiao-5 points in 60 volunteers. When both applications were completed, we asked them to write down anything that they noticed regarding the needle application and associated sensations.

**Results:**

The mean ± SD of correct/unidentifiable/incorrect answers given by the 10 acupuncturists were 17.0 ± 4.1/6.4 ± 3.6/16.6 ± 3.0, respectively. Regarding patient masking, none of the subjects commented in the questionnaire that they had received a non-penetrating needle. Of 60 penetrating and 60 non-penetrating needle applications, 48 (80.0%) and 25 (41.7%) applications elicited skin penetration sensation and 48 (80.0%) and 20 (33.3%) applications elicited *de qi*, respectively.

**Conclusion:**

These needles have the potential to mask both practitioners and patients from the type of needle used in acupuncture research.

## Background

The strongest evidence supporting the efficacy of acupuncture has been obtained using single-blind methods [1-4], which fail to meet the methodological standards for study blinding in conventional medicine [5-8]. As a result, the effectiveness of acupuncture has remained controversial, even though studies of the highest possible quality have been published in leading medical journals [8]. The reason for this is that study subjects/patients are still exposed to possible bias due to the expectations, enthusiasm, suggestions and attitude of unmasked practitioners [5-13], and placebo needles aimed at masking practitioners have been considered unfeasible [7,8,13]. Until date, there have been no published reports on the development of a procedure or placebo needle that can mask practitioners from the type of needle used.

Although single-blind trials using placebo or sham needles are a significant advance [14-16], double-blind trials using placebo needles are critically important to ensure that acupuncture research meets the methodological standards of medical science to provide stronger evidence of the effectiveness of treatment using needles [5-8]. Only then will acupuncture be incorporated into generally accepted practice [5-8,11-13].

Here, we report the design of double-blind (practitioner-patient masking) non-penetrating placebo and matched needles [17] to solve the methodological conundrum of practitioner masking [7,8,13,14] with a statistical evaluation of the masking effect of these needles.

## Methods

### Participants

We recruited well-experienced and licensed acupuncturists on the teaching staff and healthy volunteers familiar with acupuncture treatment as experimental subjects from Hanada College. Before the study, the purpose and format were explained and the subjects provided written consent. The Showa University Ethics Committee gave its approval.

### Design of double-blind needles

**Figure 1:**
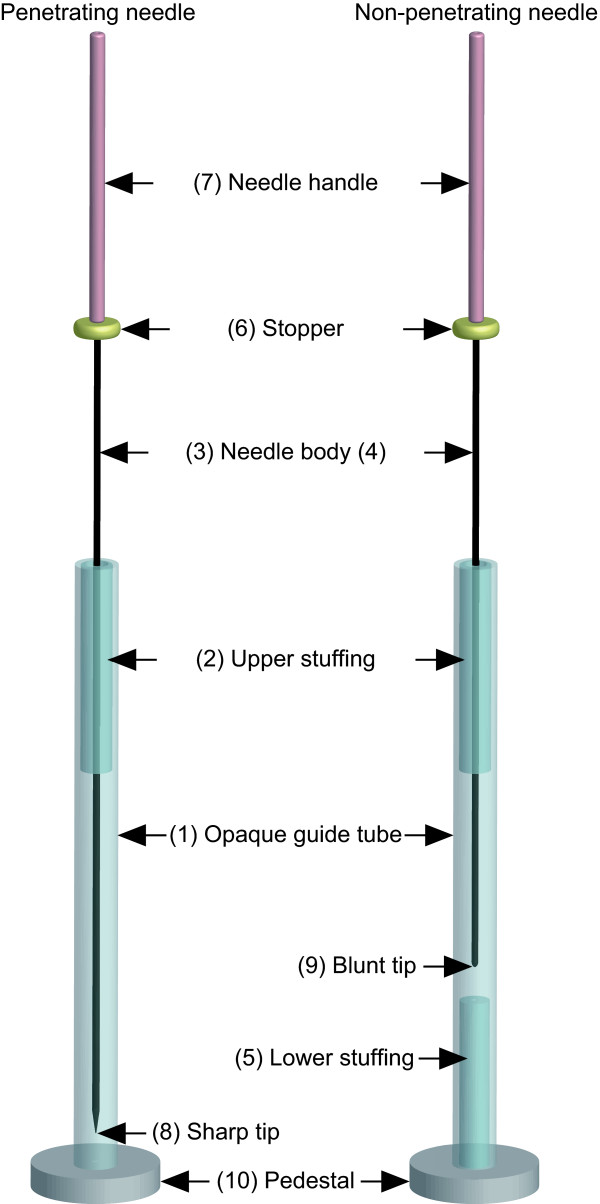
**Double-blind placebo and matched needle**. Design of the double-blind acupuncture needles. Each needle assembly comprises an opaque guide tube (1) and upper stuffing (2) to provide resistance to the needle body during its passage through the guide tube. The body of the penetrating needle (3) is longer than the guide tube by an amount equal to the insertion depth, but the body of the non-penetrating needle (4) is only long enough to allow its blunt tip to press against the skin when the needle body is advanced to its limit. The non-penetrating needle contains stuffing at the bottom as well (5) to give a sensation similar to that of skin puncture and tissue penetration. Both needles have a stopper (6) that prevents the needle handle (7) from advancing further when the sharp tip of the penetrating needle (8) or the blunt tip of the non-penetrating needle (9) reaches the specified position. The pedestal (10) on each needle is adhesive, allowing it to adhere firmly to the skin surface. The diameter of the needles used in this study was 0.16 mm.

We designed a double-blind (practitioner-patient) non-penetrating needle, the tip of which presses against the skin but cannot penetrate it, and a matched penetrating needle with a specified insertion depth to be used in acupuncture research [17]. The appearance and feel of the penetrating and non-penetrating needles were indistinguishable from one another (Figure 1).

### Validation test for practitioner masking

Ten highly experienced, licensed acupuncture practitioners (mean ± SD: 41.7 ± 8.8 years; all men) with a mean [SD] duration of acupuncture experience of 12.4 [7.8] years participated in this study (Table 1). Four sets of 10 sterilized needles were prepared as follows: one non-penetrating and nine penetrating, six non-penetrating and four penetrating, nine non-penetrating and one penetrating, and seven non-penetrating and three penetrating needles. In total, 23 non-penetrating and 17 penetrating needles were used. Before the trial began, the practitioners were informed that each set of 10 needles comprised a random number of both non-penetrating and penetrating needles. Each acupuncturist took four sets at random and consecutively applied 40 needles at the Large Intestine-4 point of author (NT), which is located between the two heads of the first and second metacarpals on the dorsal surface of the right hand [18], using the alternating twirling technique (alternating between rotating the needle clockwise and counterclockwise). The insertion depth of the penetrating needle was 10 mm [18]. Each needle was inserted and pulled out after the stopper had made contact with the top of the guide tube. Immediately after the removal of each needle, the practitioner recorded his judgment of the needle to be 'penetrating', 'non-penetrating' or 'unidentifiable'.

**Table 1:** Numbers of correctly, unidentified and incorrectly identified needles in 10 acupuncturists on 40 (23 non-penetrating/17 penetrating) needles

Acupuncturist	Years of experience	Number of correctly identified needles (non-penetrating/penetrating)	Number of unidentified needles (non-penetrating/penetrating)	Number of incorrectly identified needles (non-penetrating/penetrating)
No. 1	8	15 (10/5)	6 (2/4)	19 (11/8)
No. 2	25	21 (12/9)	7 (3/4)	12 (8/4)
No. 3	15	19 (13/6)	9 (3/6)	12 (7/5)
No. 4	13	22 (12/10)	1 (0/1)	17 (11/6)
No. 5	3	14 (12/2)	8 (4/4)	18 (7/11)
No. 6	5	13 (10/3)	12 (7/5)	15 (6/9)
No. 7	5	9 (7/2)	10 (3/7)	21 (13/8)
No. 8	25	19 (8/11)	3 (2/1)	18 (13/5)
No. 9	10	19 (9/10)	6 (4/2)	15 (10/5)
No. 10	15	19 (14/5)	2 (1/1)	19 (8/11)

Mean ± SD	12.4 ± 7.8	17 ± 4.1 (10.7/6.3)	6.4 ± 3.6 (2.9/3.5)	16.6 ± 3.0 (9.4/7.2)

### Validation test for patient masking

Sixty healthy volunteers (29.7 ± 7.5 years, 35 men, 25 women) who were familiar with receiving acupuncture were recruited for the validation study. Before the trial began, the experimental procedure was explained to the subjects as follows: 'We will apply two needles, which may or may not differ in type, at bilateral Sanjiao-5 (SJ-5) points that are located three finger widths above the wrist crease between the ulna and radius on the posterior surface of the forearm [18]. Once both applications have been completed, we will ask you for each arm whether you felt a skin penetration sensation and *de qi*, a deep dull pain sensation that is considered essential for a successful acupuncture treatment [18]. We will ask you to write down anything that you noticed, however trivial, regarding the needle application'.

Sixty penetrating/non-penetrating needle (10 mm insertion depth [18]) pairs were prepared and each needle was sealed in a small opaque container. The acupuncturist applied a pair of needles to each of the 60 subjects at the bilateral SJ-5 points, one needle in the right arm and the other in the left, using the alternating twirling technique. After each application, the subjects reported whether they felt a skin penetration sensation and *de qi *for each arm and wrote down anything that came to their notice. The practitioner guessed the authenticity of the needle after its removal. During this part of the study, the 'unidentifiable' option was discouraged.

### Data analysis

The chi-squared test (SPSS version 15.0J; SPSS Inc, Chicago, IL) was used to determine whether the number of correctly and incorrectly identified needles fitted a probability of 0.5 and to compare the frequency of needle sensations between penetrating and non-penetrating needles.

## Results

### Validation test for practitioner masking

The number of correct/unidentifiable/incorrect answers given by the 10 acupuncturists had a mean ± SD of 17.0 ± 4.1/6.4 ± 3.6/16.6 ± 3.0, respectively. Overall, the 170 correct and 166 incorrect identifications fitted a probability of 0.5 (χ^2 ^= 0.048, *p *= 0.827), excluding the 64 unidentifiable needles. Furthermore, 107 correctly identified non-penetrating needles and 94 incorrectly identified non-penetrating needles (χ^2 ^= 0.841, *p *= 0.359), and 63 correctly identified penetrating needles and 72 incorrectly identified penetrating needles (χ^2 ^= 0.600, *p *= 0.439) fitted a probability of 0.5 (Table 1).

### Validation test for patient masking

None of the subjects commented in the questionnaire that they had received a non-penetrating needle. Of the 60 penetrating and 60 non-penetrating needle applications, 48 (80.0%) and 25 (41.7%) applications elicited skin penetration sensations and 48 (80.0%) and 20 (33.3%) applications elicited *de q*i, respectively. The frequency of needle sensations was significantly different between non-penetrating needles and penetrating needles (skin penetration sensation χ^2 ^= 18.502, *p *< 0.001; *de q*i, χ^2 ^= 26.606, *p *< 0.001).

Of the 120 needles, the practitioners identified 65 (54.2%) correctly (penetrating needle = 35, non-penetrating needle = 30) and 55 (45.8%) incorrectly (penetrating needle = 25, non-penetrating needle = 30), which fits a probability of 0.5 (χ^2 ^= 0.833, *p *= 0.361).

## Discussion

The practitioners failed to distinguish between the penetrating and non-penetrating needles, regardless of their practical experience. The subjects were also unable to distinguish needle authenticity.

For double-blind acupuncture studies, real and placebo needles must be identical for all variables except skin penetration. This is the ultimate aim underlying the design of the needles; they must fit these preconditions. The appearance and feel of the non-penetrating placebo and penetrating needles in this study were virtually identical, such that even well-experienced acupuncturists required deliberation to determine whether a needle was real or placebo. The findings that 16% of all needles were unidentifiable and that the practitioners identified approximately 50% of the other needles incorrectly indicate the potential of these needles in practitioner masking. Although our results suggest that skin penetration and further insertion of the needles were masked from the acupuncturists in this study, the needles must be validated in clinical settings with variables such as clinical improvement, adverse reactions and repeat treatments with multiple needles. These variables, as well as slight bleeding and patient reaction to strong pain elicited by real needle insertion in some instances, could break the blind.

Because our masking needle is designed for use at all acupuncture points, there should be no problem in its use on the toes, fingers or scalp, which are popular sites [7,19]. It may be necessary to shave hairy skin sites to ensure firm adhesion.

Although skin penetration pain and *de qi *were less common with the non-penetrating needles than with the penetrating needles, none of the subjects suspected that they had received a non-penetrating placebo needle. This could be due in part to the fact that pain does not necessarily accompany needle insertion or removal, and that the subjects had previously experienced a very faint sensation elicited by the insertion of a fine needle. Indeed, approximately 20% of the penetrating needle applications in this study elicited neither skin penetration pain nor *de qi*. This suggests that the small proportion of study participants who did not sense needle penetration despite the insertion of a real needle might have guessed that a non-penetrating needle was being used if they had been informed of the possible use of the non-penetrating needles. Based on the findings of studies using single-blind needles [14-16], we believe that the subjects were unaware of the fact that we used non-penetrating needles due to the pressure applied to the skin by the blunt tip. However, the subjects were unlikely to report that they had received a non-penetrating needle, even if they suspected it, because we did not ask them whether they thought they had received a penetrating or non-penetrating needle. Thus, the successful subject masking observed in this study might indicate that our expectations were correct. The masking capability of the needle in study participants must be validated under conditions in which they have been informed of the possible use of non-penetrating needles, and subjects' speculations of the type of needle used must also be assessed [20]. If needle sensations *per se *can induce physiological responses, the low frequency of needle sensations elicited by non-penetrating needles would limit their use as a placebo device.

## Conclusion

The findings of the present study suggest that these double-blind placebo needles have potential for masking both acupuncture practitioners and patients. Given the recent developments in acupuncture research and practice, these needles should pave way for double-blind experiments to allow scientific assessment of the effect of acupuncture.

## Competing interests

NT and Hanada College possess the US patent 6575992B1, the Canadian patent CA 2339223, the Korean patent 0478177, the Taiwan patent 150135 and have registered the Chinese patent right (Title: Safe needle, placebo needle, and needle set for double blind) of the needles described in the manuscript. NT is a salaried employee of Hanada College and has received research funding from the college.

## Authors' contributions

NT designed the double-blind needles and the study, performed the data collection and analysis, and authored the manuscript. HY participated in the data collection and analysis, and manuscript preparation. NT is the guarantor. All authors read and accepted the final version of the manuscript.

## Pre-publication history

The pre-publication history for this paper can be accessed here:


